# General practice wide adaptations to support patients affected by DVA during the COVID-19 pandemic: a rapid qualitative study

**DOI:** 10.1186/s12875-023-02008-6

**Published:** 2023-03-23

**Authors:** Sharon Dixon, Anna De Simoni, Eszter Szilassy, Elizabeth Emsley, Vari Wileman, Gene Feder, Lucy Downes, Estela Capelas Barbosa, Jasmina Panovska-Griffiths, Chris Griffiths, Anna Dowrick

**Affiliations:** 1 Nuffield Department of Primary Care Health Sciences, Radcliffe Primary Care Building, Radcliffe Observatory Quarter, University of Oxford, Woodstock Road, Oxford, UK; 2 Wolfson Institute of Population Health, Faculty of Medicine and Dentistry, Queen Mary University of London, London, UK; 3 Centre for Academic Primary Care, Bristol Medical School, Population Health Sciences, University of Bristol, Bristol, UK; 4 Department of Psychology, Mental Health & Psychological Sciences, King’s College London, London, UK; 5 IRISi, Bristol, UK; 6 Violence and Society Centre, School of Policy and Global Affairs, City University of London, London, UK; 7 The Big Data Institute and The Pandemic Sciences Institute, University of Oxford, Oxford, UK; 8 The Queen’s College, University of Oxford, Oxford, UK

**Keywords:** Domestic Violence and Abuse (DVA), General Practice, COVID pandemic

## Abstract

**Background:**

Reporting of domestic violence and abuse (DVA) increased globally during the pandemic. General Practice has a central role in identifying and supporting those affected by DVA. Pandemic associated changes in UK primary care included remote initial contacts with primary care and predominantly remote consulting. This paper explores general practice’s adaptation to DVA care during the COVID-19 pandemic.

**Methods:**

Remote semi-structured interviews were conducted by telephone with staff from six localities in England and Wales where the Identification and Referral to Improve Safety (IRIS) primary care DVA programme is commissioned.  We conducted interviews between April 2021 and February 2022 with three practice managers, three reception and administrative staff, eight general practice clinicians and seven specialist DVA staff. Patient and public involvement and engagement (PPI&E) advisers with lived experience of DVA guided the project. Together we developed recommendations for primary care teams based on our findings.

**Results:**

We present our findings within four themes, representing primary care adaptations in delivering DVA care: 1. Making general practice accessible for DVA care: staff adapted telephone triaging processes for appointments and promoted availability of DVA support online. 2. General practice team-working to identify DVA: practices developed new approaches of collaboration, including whole team adaptations to information processing and communication 3. Adapting to remote consultations about DVA: teams were required to adapt to challenges including concerns about safety, privacy, and developing trust remotely. 4. Experiences of onward referrals for specialist DVA support: support from specialist services was effective and largely unchanged during the pandemic.

**Conclusions:**

Disruption caused by pandemic restrictions revealed how team dynamics and interactions before, during and after clinical consultations contribute to identifying and supporting patients experiencing DVA. Remote assessment complicates access to and delivery of DVA care. This has implications for all primary and secondary care settings, within the NHS and internationally, which are vital to consider in both practice and policy.

**Supplementary Information:**

The online version contains supplementary material available at 10.1186/s12875-023-02008-6.

## Background

### The primary care response to DVA

Reporting of domestic violence and abuse (DVA) increased globally during the COVID-19 pandemic [1]. While public health measures which restrict movement (such as lockdowns) play an important role in minimising virus transmission, shutting down or limiting usual routes to support and safety have a detrimental impact on the health and wellbeing of people experiencing DVA and their families. In the UK there was a reported 6% increase in DVA cases recorded by the police in the year ending March 2021 [2].

DVA is associated with physical and mental illness [3]. International guidance from the World Health Organisation [3–5] and national guidance, such as that produced by the National Institute for Health and Care Excellence (NICE) in the UK [6], recognise that DVA is gendered. A greater proportion of women than men experience abuse in intimate relationships, a much greater proportion experience sexual violence, and the violence is more severe and persistent [6]. Hence, interventions to support women have been prioritised, although services to address the needs of male survivors are also needed. Given the health impacts of DVA, this guidance has identified health systems as playing an important role in global and national efforts to support women affected by DVA, including offering non-judgemental support, practical resources and ensuring safety [4, 5].

Within health systems, international and national guidance recognises primary care as a private, safe and accessible place for people affected by DVA to access care [4–6]. The role of primary care in responding to abuse is underpinned by a broader move towards trauma-informed care [7, 8]. Primary care teams are expected to non-judgmentally address the health impacts of DVA and assess immediate safety [6]. In locations where there are local specialist DVA support services available, primary care teams can offer a route into specialist support, expanding the range of entry points for survivors[4, 6]. From the perspective of survivors, there is evidence that doctors are seen as trusted professionals to whom they can disclose [4, 9]. Opportunities to offer support can also be enabled if primary care teams find out about patient experiences of DVA indirectly through information shared via third parties (such as police or social services) or from information about abuse recorded in the medical record. In such situations, having information about DVA support clearly displayed within primary care settings enables opportunities for self-referral. However, formal disclosure in health care settings may not the preferred option for all patients, including where there are concerns that information in the medical record could be accessed by the perpetrator [10].

In the UK, the role of primary care in addressing DVA has been strengthened over the past decade, particularly through the evidence for the Identification and Referral to Improve Safety (IRIS) model [11–14]. This entails training of general practice teams in the identification, support and referral of female patients currently or historically affected by abuse from a partner/spouse or other adult in the household. The IRIS programme, which is facilitated and monitored by IRISi, a social enterprise, has been commissioned in 54 areas of the UK and has trained over 1,275 practices in England, Wales, the Channel Islands and Northern Ireland. Over 25,500 female patients have been referred from these practices into specialist DVA services in the past 10 years [15].

The IRIS programme encourages clinicians to have a low threshold for initiating conversations about abuse. Patients do not need to make an explicit disclosure of DVA or choose to use these words to describe their experiences to be referred for specialist support from their local IRIS programme, but they do need to consent and agree to the referral. This requires an open conversation and shared understanding of the service and reasons why support is being sought within it. A key component of the IRIS programme is that training is provided for the whole primary care team, including the non-clinical and administrative support staff [15]. This included adapted training at the start of the pandemic [16]. General practice teams, including both clinicians and reception and administrative staff, fed back that following training they felt confident to respond to DVA despite restrictions, but less is known about how guidance was enacted in care access and delivery during the pandemic [16].

### Covid-19 and the transition to remote consulting in primary care

The implementation of lockdowns and social distancing in the UK resulted in most medical consultations in primary care shifting to telephone or video encounters with patients [17, 18], though efforts were made to maintain flexibility in offering face-to-face appointments [19]. Consequences of this transition include some patients struggling to access primary care [20], and delaying help-seeking for symptoms, risking to presentation when ill-health was more severe [21, 22]. Where patients had successfully accessed primary care, some found communication using remote technologies more difficult [23].

There was a decline in primary care referrals to DVA services in the first year of pandemic, between March and September 2020, which was particularly acute during periods of lockdown, despite increased reporting of DVA [24]. There has been limited qualitative research into the response to DVA in primary care during the pandemic, though insights can be drawn from related literature. Dixon et al.’s study of primary care safeguarding practices during the pandemic found that clinicians worried that conversations might not be safe, and that limited continuity of care weakened safeguarding opportunities, particularly in the absence of pre-existing relationships [25]. From a patient perspective, Liberati et al.’s study of mental health servicer users’ experience of remote consulting found that some valued the convenience of remote methods when it enabled them to maintain contact with familiar clinicians. However, the absence of non-verbal cues and a ‘safe space’ for conversations were seen a barrier to building therapeutic relationships [26].

Within the literature about delivery of DVA services during the pandemic, it has been noted that survivors’ ability to seek help or communicate directly over the phone was reduced during periods of lockdown, including as perpetrators were more likely to be present or privacy became harder to negotiate [27, 28]. Increased accessibility through remote appointments, which benefited some service users, was in tension with the ability to ensure safety of remote interactions [29]. Remote support can mitigate against the need to travel to access services. However, this requires safety, privacy, and digital device and internet access, thus creating new barriers to services for others. Digital or remote interventions suited those who had consistent access to remote technologies and were digitally literate and, importantly, were able to choose to access support at a time they identified as safe [30]. Digital support can create opportunities for survivors of DVA to access care, and can offer flexibility and autonomy, but there are risks of adverse impacts on rapport and relationship building [31]. The loss of non-verbal cues and human presence are known to impact adversely on the experience of service delivery for both providers and patients [32]. These concerns raise important considerations about the potential impacts of digital and remote care on equity of service provision and access to care.

Taken together, this literature highlights the importance of safety, accessibility, and continuity of care in the transition to remote consultation in primary care for vulnerable patients. While pragmatic guidance was produced early in the pandemic [33], no studies have reported how primary care teams have adapted practice in relation to patients experiencing DVA, addressing the challenges of maintaining safety and confidentiality. This is important when initiating remote conversations about abuse and facilitating trusting patient-practitioner relationships. Moreover, there is limited understanding of how primary care teams work collectively to identify DVA.

In this paper we address this gap, examining adaptations made by primary care teams that transitioned to remote consultations during the pandemic to continue creating opportunities for patients to disclose DVA. We aimed to understand the experiences of primary care clinicians, practice management and administration teams, and specialist DVA staff (IRIS Advocate Educators) responding to patients affected by DVA during the pandemic, examining this as collective work. Our analysis forms part of a wider study of the impact of the pandemic on the primary care response to DVA [34].

## Methods

### Sampling and recruitment

This was a qualitative study using semi-structured interviews conducted remotely (via phone or video call). We used a multi-stage sampling framework, as described in our study protocol. In stage one, in partnership with IRISi regional managers (who are responsible for supporting the local commissioning and delivery of IRIS), we identified a geographically and socio-economically diverse sample of areas where the IRIS programme was running. This process identified ten areas which were invited to participate and six of which agreed. As stage two, Advocate Educators (AE) in each area suggested practices to approach for interview participants, informed by our goal of representing diverse practice structures and population demographics and variable engagement with the IRIS programme. From these initial contacts we conducted snowball’ recruitment of individuals within general practices and AEs involved in the IRIS programme. Sampling decisions were guided by insights from IRISi regional managers (see the PRECODE Protocol for details, including inclusion/exclusion criteria) [34].

[1] Between April 2021 and February 2022, we conducted twenty-one interviews with eight GPs, three practice managers, three reception/administrative staff, and seven IRISi AEs from four cities in England and Wales. Please see Table 1 for participant demographics. To maintain participant anonymity, we numbered their contributions sequentially: GP for general practitioner, PM for practice manager, admin for members of the administrative and reception teams, and AE for Advocate Educator (e.g., GP1, AE2).

**Table 1:** Participant demographics

Characteristic	N (%)
**Type of healthcare professional**	
Advocate educator (AE)	7 (33)
GP	8 (38)
Practice manager (PM)	3 (14)
Reception team (Admin)	3 (15)
**Gender**	
Female	20 (95)
Male	1 (5)
**Years spent working in sector**	
0–3	2 (10)
4–9	9 (43)
10–20	2 (10)
20 +	3 (14)
Not specified	5 (24)

### Data collection and analysis

Semi-structured interviews focussed on experiences of managing DVA in primary care during the pandemic: views about the utility of online DVA training, exploring concerns with and experiences of asking (or not) about DVA; relevance and availability of guidance; obstacles to and strategies for offering support and referral (see Appendix A for topic guides). Interviews were conducted remotely, via phone or MS teams, by EE, ADS, AD, VW, and SD. Interviews ranged in length between 12 and 69 min and audio recorded, transcribed verbatim, anonymised, and checked for accuracy.

We applied rapid qualitative analysis techniques using a data-driven inductive approach [35, 36]. Interviews were initially summarised within rapid assessment procedure (RAP) sheets (ii), which were reviewed regularly within the research team and were transcribed verbatim. The qualitative research team (EM, ES, AD ADS, VW, EE, SD) met frequently (fortnightly) to review the RAP sheets and interview transcripts. The qualitative team all read and familiarised themselves with each interview RAP sheet and transcript. ADS and SD coded the interview transcripts using a framework approach. Through discussion and iterative cross comparison of the data, we utilised mind-mapping to identify themes and sub-themes and to consider how and where these might be inter-related [37]. To ensure transparency and trustworthiness, our developing analysis and identified themes were reviewed regularly within the wider study team, and with our study PPIE advisers, who assisted in developing and sense-checking our analysis.

### Patient and Public Involvement and Engagement (PPI&E)

This project has been guided by a group of female PPI&E advisers, with lived experience of accessing services for DVA. They were consulted regularly at meetings throughout the project. They advised and informed our research approach, research questions, and how we could utilise what we have heard to support general practice access. Based on our findings, we worked with them to develop recommendations which primary care teams could consider when caring for patients affected by DVA (see Discussion).

### Ethics

The study received HRA (Health Research Authority), Health and Care Research Wales (HCRW) Approval (20/HRA/5873) and University of Bristol Faculty of Health Science Research Ethics Approval (113044).

## Results

We present our results under the following thematic headings: 1) making general practice accessible for DVA care 2) general practice team-working to identify DVA 3) adapting to remote consultations about DVA, and 4) experiences of onward referral for specialist DVA support. These are summarised in Table 2

**Table 2:** Themes and sub-themes

Theme	Sub-themes
Making general practice accessible for DVA care	Encouraging patients to come forward to discuss DVA
	Interface with external media and communications
	Adapting triage processes for appointments
General practice team-working to identify DVA	Reception and administrative teams recognising DVA
	Collaborating within the practice to identify DVA
	Using external information to identify DVA
Adapting to remote consultations about DVA	Arranging consultations safely
	Identifying cues for discussing DVA remotely
	Transitioning from remote to face-to-face to facilitate disclosure
Experiences of onward referrals for specialist DVA support	

### Making general practice accessible for DVA care

Ensuring that patients affected by DVA could safely access general practice was a challenge during periods of reduced access to services, particularly lockdowns. Patients affected by DVA were perceived as less likely to seek help, so staff undertook activities promoting their willingness to provide support for DVA, such as posting on social media. Practices also adapted triaging processes enabling access to face-to-face consultations, given that patients were less likely to be able to disclose DVA over the phone.

#### Encouraging patients to come forward to discuss DVA

Concerns about the impact of the pandemic on DVA among participants focused on the impact of infection control adaptations during the early pandemic period, when all initial contacts were undertaken remotely by telephone or online consultation. GP6 recalled anxieties that patients would not come forward about DVA during the pandemic, in part because of concerns about infection, and there were concerns about public safety messaging having a potential adverse impact on people feeling able to contact health services:

*Just the absolute fear of people not coming forward about domestic violence, and also their increased risk of domestic violence during the pandemic. We have all been really concerned about that. And the concerns at the way we have been asked to consult. And the fact that people were told not to bother their doctors about things was my main concern GP6*

One support worker (AE1) explained that the fear of being isolated or alone, including when unwell, might influence whether patients would access support:

*People that would normally think, “Right, actually, this is becoming quite risky because you are not acting in a way I would want, and the children are seeing things they shouldn’t do.” But it is that fear of being on your own as well because people are quite anxious because of COVID* (AE1)

Strategies that practices used pre-pandemic to signal their willingness to support patients experiencing DVA, such as displaying posters, were compromised during the pandemic. Recognising this, some practices adapted their strategies, for example adding information to practice websites or social media to offer support and information for those experiencing DVA.

*I remember guiding our admin staff to post lots of information on our Instagram page, we updated our website with lots of DVA information on it. So, at any point along the way, at least it was visible, what to do, where to go, how to contact...replacing the waiting room information* (GP2)

#### Interface with external media and communications

Conversely, participants discussed how widespread media coverage about the escalating rates of DVA during the lockdown might promote care-seeking from those affected and awareness in primary care. This was in addition to public encouragement for people to contact their general practice if suffering from DVA.

*… I know it's because, as I say, they advertise in the media, TV, GP series [TV programme about GP practice], about abuse and they're maybe identifying it themselves... I think doctors are thinking more about it because it's been on the news that it's more likely to be happening.* GP7

#### Adapting triage processes for appointments

For many practices, a significant change to their working practices during the early stages of the pandemic was the requirement to move to ‘total triage’. Patients were asked to give a reason for their consultation via phone or online and then allocated either a telephone, video or face-to-face appointment. Participants reflected on the implications of this policy on access to care, including whether patients would be able to participate safely in this screening process.

*We had to divert to online consultations as well where they would fill in a form. And, again, if somebody is being controlled at home, they’ll have access to that. So, they couldn’t be honest in those forms either. So, we would never know unless somebody walked through the door really* (PM2)

Staff recognised that patients might not feel safe or comfortable to disclose that they wanted an appointment to discuss DVA. Instead, they might present with a different issue. This made it difficult to ensure that patients who needed privacy were offered a face-to-face appointment.

*But in relation to the domestic violence, a lot of people that perhaps used to come to the doctors with a minor thing to, kind of, create a situation where they could disclose something, they couldn’t because… We were doing telephone triaging and we were doing telephone consultations, but their partners tended to be there. So, they couldn’t say what they needed to say.*

*So they might have come in because of their bad back and then you end up talking about, you know, historical or contemporary domestic abuse or domestic violence. There isn’t the same opportunity for that to occur in lockdown because you are not sitting there in the room, in a place of safety.GP1*

This triage process also included COVID screening, where anyone with respiratory symptoms would not be able to come into the practice. Some staff were concerned this could prevent those in need accessing a face-to-face appointment, particularly if physical symptoms were a ‘cover story’ to gain a face-to-face appointment.

*And they might come up with, “Oh, I’ve got a cold.” We would just say straightaway, “Well, you can’t come in.” Because it could be COVID. But that could be the cover story and the receptionist is not going to know that and neither is the doctor PM2*

Asking why someone wanted an appointment could act as a barrier in accessing appropriate care if a patient was unable to safely disclose DVA as a reason. Some practices improvised solutions where, if DVA was flagged on their record, patients would, by default, be invited for a face-to-face appointment. An advantage of the COVID rules was that it became easier to see patients alone. This could help create private spaces for care:

*The other good thing with the pandemic is though, that you can actually without causing too much alarm or upset, you can ask the partner not to come in.”* (Admin1)

### General practice team-working between clinicians and administrative staff to identify DVA

Teams worked together to identify patients who might be affected by DVA. The role of reception and administrative teams in identification, both in noticing signs of abuse and collating information from external bodies (for example, letters about safeguarding processes, from the police, or from other health providers, such as emergency departments, health visitors, or midwifery colleagues) about DVA, remained vital during the pandemic, including managing the initial point of contact. Training about DVA adapted to reflect the challenges of the pandemic supported this.

#### Reception and administrative teams recognising DVA

Practice teams often worked collaboratively to identify patients experiencing DVA, drawing on the opportunities to observe signs of abuse or highlight external information.

While the pandemic limited the number of patients physically visiting the practice, unusual behaviours were noticed by receptionists and administrators who could then share them with the wider practice team. Participants spoke about a range of situations which might prompt them to raise the issue of DVA with colleagues, including patients not picking up prescriptions, missed appointments, unusual telephone conversations, and seeing or hearing partners or family members exhibiting controlling behaviour.

*Because this young lady didn’t pick her prescription up on the one week, her weekly prescription…And she was highlighted as a very at-risk patient, for historical domestic violence….[] So, I relayed that back to the doctor and she went and did the visit. And she has actually gone into a refuge now, a fortnight ago. Admin 3*

The ability of receptionists and administrators to notice and act on these behaviours was facilitated by training, awareness, and preparedness. All participants in this study had received training in recognising and safely responding to DVA. The AEs involved in training recognised the importance of the non-clinical roles.

*It starts with having the IRIS information, the website, having the posters up, so creating that… You know, making sure that the admin staff are trained around domestic abuse to create that practice that becomes a safe space. Initially, when the woman walks in, she can see that, “Okay, this is a place where I can talk about domestic abuse.”* (AE2)

#### Collaborating within the practice to identify DVA

Before and during the pandemic, regular discussion of potential DVA cases at practice meetings enabled bringing together diverse observations and identifying ways to offer support. This could include opportunities to discuss external information that had come into the practice or initial contact encounters where the reception teams wanted to share uncertainty or seek advice. These also created opportunities to highlight patients potentially affected by DVA to the wider practice team.

*We feedback to each other. And it might be that nothing comes of it…. So, everybody in the practice is aware of that person. So, if they do ring up, we’re more aware, the girls on reception would be more aware of that phone call and would perhaps not be dismissive of it and try and treat it a bit differently to others.* (PM2)

Some reception and administration participants felt there was better collaboration between themselves and clinicians.

*The doctor actually sends us messages, to say, “Keep an eye on these names.” So, they’ll send us a message to, if we see anything, let them know. So, we’re working more together now.* (Admin3)

#### Using external information to identify DVA

Reception and administration teams’ process information communicated to the practice, a critical aspect of supporting patients affected by DVA. Information about DVA can enter primary care from a number of routes including other health settings (e.g. emergency services, sexual health services, ante-natal clinics), police, safeguarding teams, and in medical records of newly registered patients. These records can include references to both historic and recent experience of DVA.

Receptionists and administrators told us how documentation of DVA in patient notes, including alerts, could be used to guide both triage requests for clinical consultations and help the team consider how they could offer safe access to care and support.

*Upstairs in the offices they’re pretty good at picking up on the latest information that’s out there and then kind of passing it down, and then we kind of adapt it, we have sort of weekly meetings with management, with the leads and things like that and we discuss how we’re going to do it or what we can do. If it was something domestic violence related, we would just give them a same day appointment.* (Admin 1)

Practice managers, who had oversight of information flows and internal alert systems, were key actors’ in ensuring that DVA could be safely recorded on patient records.

*[T]hat is how the practice does it. They ask to put the information on that manage button so everyone in the practice is aware, so if they ring up for an appointment you can see, “This patient could be in danger of domestic violence. Please make sure you give an appointment,” so they get an appointment that day. But they wouldn’t be fobbed off. They wouldn’t be told, “No, we haven’t got anything for you.” We would ask the duty GP and get them in on that day. PM1*

### Adapting to pandemic consultations and recognising DVA

During remote consultations, without visual cues, clinicians found it more challenging to recognise the possibility of DVA. Most disclosures were received when an appointment transitioned to face-to-face.

#### Arranging consultations safely

Staff who had the opportunity for a face-to-face consultation could utilise these to enable conversations about DVA. Participants described how ‘disclosure is not a one-time event, it’s a process’ (AE2). While the clinical consultation was usually identified as the epicentre of the process of DVA care, the work of creating the possibility of disclosure represented a whole team effort, including preceding clinical and non-clinical encounters and actions.

Clinicians’ pre-pandemic experience made them aware that conversations addressing DVA can be sensitive and complex, requiring trust and rapport. For those with experience of receiving disclosures, their skills had largely been developed in the context of face-to-face consultations ‘where a patient feels safe, there is no one else in the room*’* (GP1).

Transitioning to the telephone (or video) necessitated navigating additional practical and safety considerations, including establishing whether the patient was alone, able to speak freely, and who else might be listening, watching, or reading emails.

*You are talking to someone on the phone and there might be an abusive person in the background. How are you as the patient going to reveal something to the GP? Where is the trust, where is the security? So, I think this has all changed. The opportunities to detect abuse and violence have been diminished* (GP1)

A strategy which teams used to address this concern was asking initial questions about safety and privacy, using the flexibility of phone consulting to arrange mutually acceptable times for conversations.

*I actually said to the woman, “Just tell me a yes or no answer. Are you able to speak?” and the answer was, “No.” It was like, “Right, okay. Let’s arrange a time when you think you are going to be on your own, that you can speak.”* (GP5)

#### Identifying cues for discussing DVA remotely

The lack of visual cues during telephone consultations reduced opportunities to start conversations about DVA. Some participants reflected that this was ‘slightly easier on video’ but safety concerns remained as ‘you don’t know who else is in the room’ (GP6). Being unable to see and respond to body language and facial expressions made it harder to both identify unexplored concerns and to demonstrate empathy.

*I found myself having to be a bit blunter in some ways in the questioning. Because patients have not really picked up on my non-verbal communication either. So, yes, it has been tricky, harder to pick up on the cues and harder to bring them up.* (GP5)

Participants described having to rely on tone of voice and ease of interaction as a source of cues about possible abuse.

*You’re looking at the tone of the voice. Do you feel that they’re not answering your questions in an easy manner? Is somebody in the background telling them what to say? Is what they’re saying quite almost short answers that they just seem to be not easy with discussion, that you’ve got a feeling that just, something isn’t quite right because obviously, it’s difficult on telephone*. (GP8)

These challenges could result in consultations being experienced as more transactional, focusing only on the presenting problem. It was harder to identify ‘hidden agendas’ (GP1) that may underlie the clinical presentation. The difficulty of noticing cues over the phone meant some practitioners were less likely to ‘go the extra mile’ (GP5) to start conversations about DVA with patients.

*Not that I am saying we would ever purposely ignore bad or domestic violence, but like I said, do we end up going that extra mile to that person that really needs it?* (GP5)

While remote consultations and DVA disclosure were often difficult, there were concerns that some patient groups were particularly disadvantaged by remote consultations. Patients with hearing impairment, learning disabilities, or language or technology barriers (including the costs of data, access to internet and private devices) were identified as particular concerns.

*[T]he groups that don’t speak English. For me, it’s pretty impossible, if I’m honest. By the time you pick them up as a patient and sorted out a telephone interpreter, you’re already probably, 20 minutes into the consultation. Then, you’ve got to then speak to them through the interpreter.*

*They, for me, would be very, very difficult. Even as a doctor, to have the patient centre resolve what they’ve actually rang up for, pick up the cues, question more on that. It’s a very patient doctor, who’s got a lot of time on their hands, that can push on that. [] houses that are full, jam-packed, of family members. When are you going to get a room, a quiet room where there’s nobody else in the room when you’re talking to them? You’ve got a family member that’s there in the background, interpreting and you’re trying to talk on their behalf. It’s difficult GP8*

#### Transitioning from remote to face-to-face to facilitate care

Most DVA disclosures received by participants during the pandemic were made when clinicians moved from remote contact to a face-to-face appointment. While some of these face-to-face consultations were prompted by concerns about DVA, often they were based on triaging protocols that differed between practices. While many prioritised face-to-face consultations for physical examinations, others discussed the potential opportunities to explore DVA within appointments for mental health problems. This could be factored into triage decisions about location of appointment.

*For example, if a patient calls about feeling overwhelmed or mood, then we always ask about, “Who is at home with you? […] “Who do you live with? […] If they say, “I live with a partner,” I just, very non-judgmentally, say, “And everything is okay between you and your partner?”* (GP3)

*I am just talking about those no answers or those subtle cues for patients who have not called to report that, but that is something on their mind. Maybe those are the ones which might not divulge it as much. (GP3)*

Even when clinicians perceived it was safe to discuss DVA over the telephone, they would often arrange a face-to-face follow-up, recognising that even when a referral had been arranged, they may still have additional needs or concerns that would be better explored face-to-face.

This could include creating strategies to bring women into the GP practice to ensure a safe and private setting to explore any needs or concerns:

*[I]f I was suspicious of DV, they'd be coming in. And I would be discussing and supporting, if need be, reviewing every week, depending on the level of risk…. So I just said, “If anybody asks you, it's for a female examination. And I can't do that over the phone, and I can't do that visually. So, I can check, I think it's best you come in. Certain clinical conditions cannot be done on the phone.”GP2*

When patients seek to discuss DVA in healthcare settings, disclosure of abuse is easier in the context of a trusted professional relationship. Staff found that having a pre-existing relationship with the patient or a previous record of DVA, could partially mitigate barriers of remote consulting and facilitate conversations about DVA.

*I’ve been a GP at this practice for a few years now. Some of the people, I may have already known and know a face due to previous consultations pre-pandemic. That, in terms of rapport, is easier than if you’ve never, ever, ever, laid eyes on them and never interacted with them before.* (GP4)

Disclosures, usually made to clinicians, were also made to other practice team members. This included non-clinicians who offer a range of advice and services to patients. These encounters created new spaces or opportunities for DVA disclosures:

*I said to her, “How are you getting on?” She said, “My benefits came through.” Then she burst into tears and said, “But he took all my money.” “Threw coffee at me”. So, then I had a word with [name of GP] and it was a referral, and it was different other things. … []..before she may not have said anything, because she might have just- she probably would have thought, “How can I go to the doctors and ask for that?”* (Admin 1)

Although many remote consultations occurred on the phone, clinicians were usually still working in their practice building. Sometimes, including working from home when isolating, GPs worked away from their practice. This could mean that as well as being remote from patients, they were remote from their colleagues and distanced from professional support within the GP team. Although flexible working arrangements were enabling in some ways, the missed opportunities for colleague support could lead to a sense of professional isolation.

*[A]s a GP, remote consultation is lovely. It’s a great change to my week and my working week, around the kids and all that kind of stuff. It’s fabulous. You don’t feel part of the practice, necessarily. You’re quite isolated on your own. So, you probably don’t have the same chats with your colleagues and all that kind of stuff, that you would normally do.GP8*

This was particularly significant for staff working for national services such as NHS 111 or digital remote consultation providers, supporting patients anywhere in the country without knowledge of local services, access to medical records, or opportunities to invite patients for face-to-face consultations. This was identified as a substantial gap in training and resource information:

*There are doctors all over the place now, which has been really promoted because of what happened over COVID, that are now working remotely and there’s none of the training. There’s nothing, at all, to do with domestic abuse. If you think of how hard it is to know what cues you’re looking for and what you do and what you do say and what you don’t say and all that kind of stuff. I think it’s just missing a massive, massive, huge proportion of remote workers now, that haven’t had that training* (GP8)

### Experiences of onward referrals for specialist DVA support

Among participants who had access to referral pathways into specialist services via the IRIS programme, access to DVA support and services following disclosure was relatively unchanged during the pandemic.

*Whether it’s pre-pandemic, mid-pandemic, or post-pandemic, it is just fill in a form, get it sent across.* (GP4)

Once a referral had been made, IRIS AEs offering specialist support explained that their use of remote models of care were effective in enabling patients to share their stories, sometimes quicker than expected.

*We’ve found that quite a lot of people are disclosing [to support services] more quickly. […] So, they are telling us more and they can do that sooner on in the process... Because we are remote and because it’s easier for people to be up-front about stuff, I think we have had a lot more disclosures of sexual abuse and serious physical abuse, sooner in the process than we normally would* (AE4)

Fears of isolation and illness during the pandemic made accessing care or leaving abusive relationships harder for patients supported by IRIS AEs. One potential consequence of this was that the referrals and presentations that were made were ‘later’ than they might have been or had become high-risk situations.

*By the time that they do report the abuse has it got to the point where it is high risk when they are at the point where they think, “No, actually, something horrendous is going to happen here”* (AE1)

## Discussion

### Summary

Our study was based on interviews with general practice teams and DVA support workers and focused on how the general practice response to DVA changed during the restrictions on patient contact mandated by UK Government COVID policy. Our study articulated how a team-based approach underpins effective primary care pathways to DVA support: from reception and administrators, to clinicians, and the specialist support services (AEs) who receive referrals. We examined the collective work of identifying DVA and enabling opportunities for safe conversations about DVA. This team-based approach was challenged by the sudden shift to remote interactions with patients during the pandemic and remote working by clinicians. Participants reflected on how this change affected their ability to notice signs of DVA, create safe opportunities for conversations about DVA with patients, and refer patients into specialist support.

**Fig. 1:**
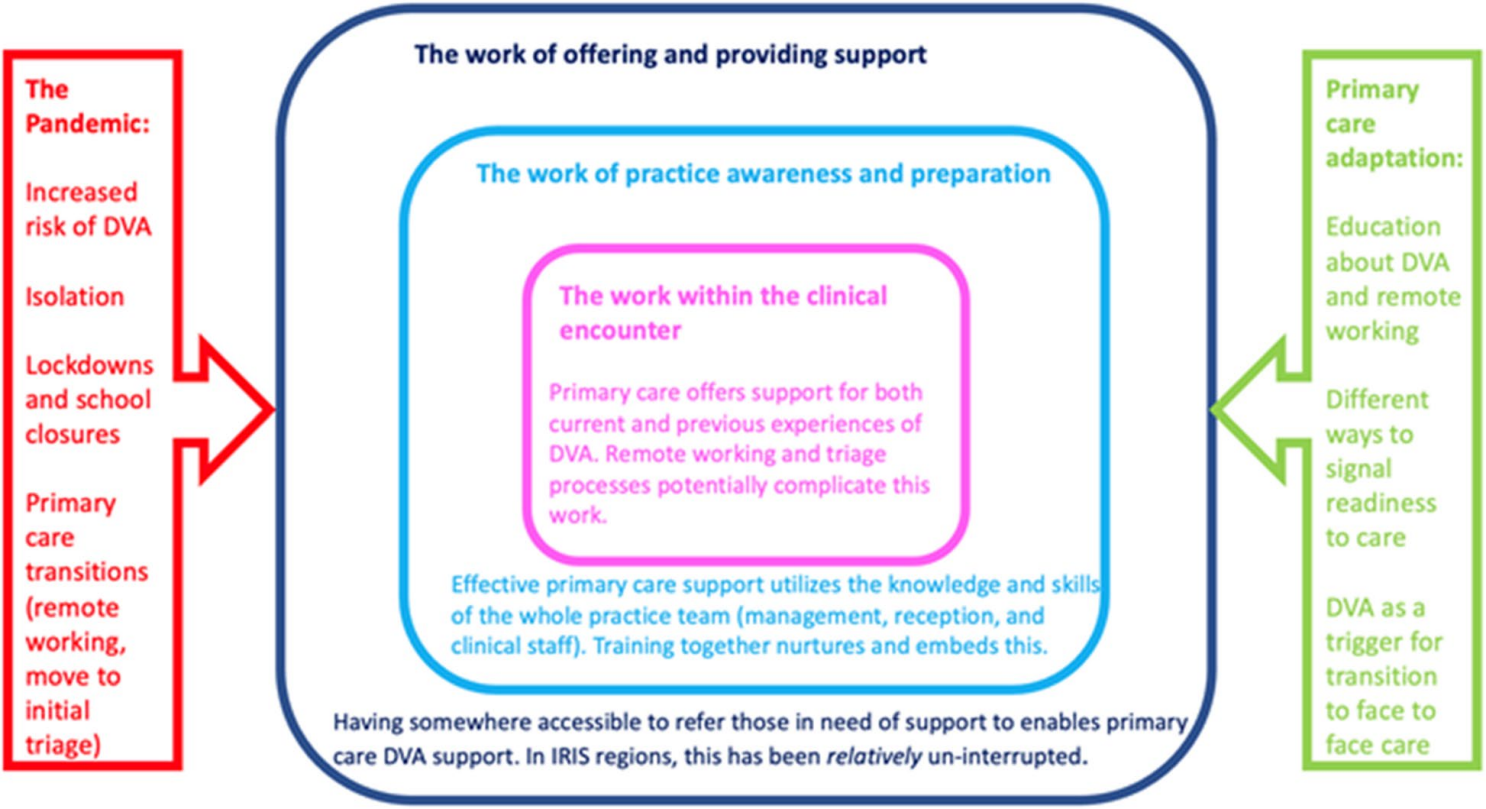
General practice team-working to provide DVA support during the pandemic

We have developed a model (Fig. 1) to represent our findings about how primary care teams worked together to support those affected by DVA during the pandemic. This situates the clinical encounter within the context of the wider practice team, demonstrating how their work is under-pinned by access to external services including pathways to referral and support and training.

### Comparison with existing literature

In previous work we have shown that referrals to DVA services from primary care fell during the pandemic, with the reduction greatest during the lockdown period [24]. In this paper we report the experiences of primary care teams and DVA support workers that could help explain this reduction, including difficulties in accessing safe consultations during the pandemic. However, we have also identified opportunities for proactive care and team working that potentially mitigates these difficulties.

There is relatively little literature examining whole team perspectives on delivering care for those experiencing DVA. McGarry et al. explored the value that teams being trained together adds to care, including awareness of DVA by receptionists at the front desk [38]. Our study expands on those findings, contributing insights about how training can enhance collaborative working to support patients, particularly in the context of (pandemic-related) changes in practice. Collaborating within a team was a key finding of a qualitative meta-synthesis exploring health practitioners’ readiness to tackle DVA [39]. This review also identified the value of having appropriate services for onward referrals, and a supportive professional culture that enabled clinicians to de-brief with each other and specialist professionals [39]. Our study widens the understanding of how the primary care team enables effective responses to DVA.

Pre-pandemic, it was known that within the clinical encounter, continuity of care is an important component of developing the trust and rapport necessary for effective DVA care [40, 41]. Our study shows how this remained true in the pandemic setting, including remote consulting and triage. Conversations about DVA were more common in face-to-face appointments when clinicians and patients had a pre-existing relationship. Both remote consulting and triage are likely to remain part of UK primary care [42] meaning that these findings remain relevant.

Continuity of care also has informational components. Managing third party information is an important part of enabling DVA care in primary care, albeit with challenges around ensuring safety and privacy of information [43]. Our study shows that when external information about patients who have disclosed that they are experiencing DVA in other situations, for example, in an emergency department, or to social workers or police, is shared with general practice teams, then this information can be used to facilitate care including face-to-face consultations in practices adapting to total triage.

Many of our findings align with research from other settings exploring professional perspectives on the interface between remote consulting and care for patients and families experiencing DVA. Concerns about the safety of phone and remote encounters have been documented by practitioners in other settings [44], as have concerns that these consultation modalities impact adversely on interpretation of cues, on developing trust and rapport, and on assessing risks and safety [25]. Taken together, these can lead to a perceived loss of effectiveness of the therapeutic encounter, whereby these consultations are experienced as shallow, with a narrower focus [25, 26, 45].

In their survey of Australian social work care providers’ experience of adapting their services to support patients affected by DVA, Cortis et al. reported the challenges of the loss of cues and safety risks but welcomed the flexibility of remote and telephone working in being able to navigate safe times for care[29]. Being able to use the greater flexibility these new ways of working offer to enable safe and equitable care for those in need is a potential positive learning point for practices [25]. Throughout the literature, there are pervasive concerns about those who might be restricted in how and when they can access care settings because of digital poverty (for example lacking devices or internet access), alongside exclusion because of language or other barriers to care [25, 45].

Finally, we identified the toll that the stress of holding DVA concerns and working remotely can have on practitioners’ wellbeing. This is echoed in research with English GPs, and in other settings [25, 45].

### Strengths and limitations

We were able to speak to the wider team of practitioners, both clinical and non-clinical, throughout the care journey from seeking an appointment to referral for DVA support. Uniquely, we have articulated whole team perspectives and demonstrated how the clinical encounter can be situated within practice processes in the context of pandemic adaptation. We sampled from socio-economically and geographically diverse regions and areas with different historical referral rates to IRIS.

A limitation of our study was the exclusive focus on practices that were part of the IRIS DVA service and training programme. We are aware that our participants may have had specific interests or expertise in DVA care, introducing potential participation bias. Therefore, these findings are not representative of all practices or practitioners. However, we consider that their insights into how the consultation is embodied within the wider practice team offer an arena for reflection for all primary care settings, both in the UK and worldwide.

### Implications for practice and research

This study does not represent the experiences of patients seeking to access primary care for DVA support during the pandemic. While it is valuable to reflect on the learning of practitioners about cases where DVA needs were identified by their teams, there is a pressing need to also learn from patients when this did not happen and where care was not enabled. Further research among people seeking help from primary for DVA during the pandemic is required.

To translate our study findings into practice recommendations, we collaborated with our study PPI experts who have lived experience of DVA. They valued the approach of developing and supporting a whole team integrated approach to DVA care, and this resonated with their experiences of safely accessing care. We co-produced a guidance based on our findings that primary care teams could consider in their training and work to support patients affected by DVA. These should be implemented alongside access to effective, accessible, and safe specialist care and services ( Table 3).

**Table 3:** Guidance for practices about DVA disclosures in remote consultations from PPI group

•It is vital to establish whether the person can speak freely and safely; have a low threshold to arrange to speak to people alone, including arranging repeated calls or face-to-face appointments at a time which the person chooses
•You can create non-verbal opportunities for help-seeking, for example having posters in reception areas and consulting rooms that patients can look at to signal to a member of the GP practice team that they have a need for a conversation at a safe time
•Access to primary care consultations can be difficult to negotiate. Consider accepting a simple request such as ‘a need for a face-to-face’ appointment, without questioning. Requiring requests for appointments in writing, including through online triage, can be a barrier to care, as this may not be safe or private for the person
•While recording DVA in medical records can be an important tool for promoting safety, this is not without potential risks and complications (e.g., perpetrators trying to access victim’s medical records). Practice team awareness of these risks and the development of safe and confidential strategies and systems when communicating as a GP team are essential
•Kindness, and developing trust and rapport enable care for people affected by DVA; continuity of care supports this and can be actively nurtured

## Conclusion

Research into DVA identification in health care settings tends to focus on the consultation or clinical encounter as the epicentre of the process of DVA disclosure. Disruption caused by the shift towards remote consulting during the pandemic has provided an opportunity to examine how the processes and context around the consultation itself are an equally important part of the process. Within the consultation, working remotely from the patient complicates DVA conversations, because of concerns about privacy, safety and confidentiality, challenges in developing rapport and demonstrating empathy, as well as the loss of visual cues. Whole team processes and collaborative working can support identification and care, including creating and supporting practice procedures and meetings which facilitate sharing information. This has implications for the evolution of remote primary care delivery. There needs to be support, resourcing and education about new and re-occurring DVA for primary care teams.

## Supplementary Information

**Additional file1**.

## Data Availability

Given the highly sensitive nature of DVA research, we will adopt trauma-informed data sharing approaches, still consistent with open science. Anonymised transcript (if safe and appropriate) will be stored on the University of Bristol’s Research Data Service Facility. Bona fide researchers will be able to access non-identifiable data upon reasonable request. Access will be subject to a data access agreement and following approval from the Chief Investigator (Professor Gene Feder, gene.feder@bristol.ac.uk) and the University of Bristol Data Access Committee.
