# Supplementary material for: General practice wide adaptations to support patients affected by DVA during the COVID-19 pandemic: a rapid qualitative study
Source: BMC Prim Care. 2023 Mar 23;24:78. doi: 10.1186/s12875-023-02008-6 (PMC10034249; doi:10.1186/s12875-023-02008-6)
Supplement: Supplementary file 1 — Additional file1. [file 12875_2023_2008_MOESM1_ESM.docx]

**Appendix A – Topic guides**

**Topic Guide 1 – General practitioners**

In general, what role do you think GPs play in supporting patients affected by DVA?
Before the pandemic, what was the usual process of supporting patients who disclosed DVA?

In general, how has the pandemic impacted your day-to-day work as a GP?

How have these changes impacted the support you provide to patients experiencing DVA?

Have you received any support or guidance about how to support patients experience DVA during the pandemic? If so, how useful was it?

Has your practice adapted or reorganised any processes of work in relation to DVA or safeguarding?

**Part 2: Identifying DVA during the pandemic**

Before the pandemic, how did you approach conversations with patients about DVA? Has this changed as a result of the pandemic? If so, how?

Have you had any conversations about DVA with patients during the pandemic? In your experience, are women disclosing DVA? When/why/has this changed? How are they letting you know that they are experiencing DVA?

If yes: What promoted the discussion? Was this different from discussing DVA before the pandemic? If so, how? How have the changes in general practice during the pandemic?

If no: In what scenario might you initiate a discussion at the moment?
What is different about approaching DVA conversations now compared with before the pandemic? How might the changes in general practice during the pandemic (e.g. the issues identified earlier) impact on your ability to discuss DVA?

In what way does remote consulting affect conversations about DVA? Consider the safety of phone conversations, explore experiences and how challenges overcome.

Are there any patient groups it is easier to talk about DVA with via remote consultation? Any groups that are harder? Why do you think that is? Is/how is this different with women you knew before the pandemic/known DVA? Is this different for new relationships/new concerns?

As a practice team, have you approached identifying DVA differently? Have you discussed concerns about patients you suspect may be affected by DVA?

Thinking about an instance in the past in which you felt you had a successful conversation about DVA, in what way would that have been different if the consultation had been remote?

Going forward, what changes would support conversations about DVA during remote consultations?

**Part 3: Providing support to patients affected by DVA during the pandemic**

Before the pandemic, what forms of support did you provide to patients affected by

Ongoing emotional/practical support in general practice did you work with the IRIS service to support patients?

Has the pandemic changed the way in which you support patients affected by DVA? How? Do you think this is the same for colleagues across general practice?

Has the level of risk changed? Are women presenting sooner or later with DVA?

Have you offered support to any patients during the pandemic? If yes, how was your experience of this?

Has the referral process to IRIS changed during the COVID-19 lockdown and ongoing pandemic restrictions, and how?

If you have made a referral:

- -  Have you received feedback?
- -  Have you seen the patient again since referral?
- -  What do you see as your role with the patient going forward?

If hasn’t supported a patient: how would you feel about referring a patient for support at the moment? What are the processes?

In general, how have you found interactions with the IRIS service during the pandemic?

Have you received support for addressing DVA from any other colleagues or services (e.g. police, social services)

What other support would help GPs to engage with patients about DVA?

**Part 4: IRIS training during the pandemic**

Have you received training from the IRIS team previously?
If yes, which training? (e.g. session 1/2/refresher?)
How was the training? What, if anything, did it change about your understanding of DVA? Was there anything else you would have liked included?

Have you received any online training from the IRIS team?
If yes, how was this? Were there any practical issues?
What, if anything, did it change about your understanding of DVA? Was there anything else you would have liked included?
(if participant has received face-to-face training from IRIS before) How was it different? Was anything better/worse?

**Summary question:**

To summarise from our discussion, what would you say has been the main impact of the pandemic on your work in relation to DVA?

**Topic Guide 2 – Advocate Educators**

**Part 1: General introductory questions**

Before the pandemic, what were the usual process for training GPs and supporting service users?

In general, how has the pandemic impacted your day-to-day work as an AE?

How has the pandemic impacted on referrals from GPs? In your experience, has there been any change in the types of questions GPs ask you? Or any change in them asking for advice/raising concerns?

Have you noticed any change in the types of referrals you are getting from GPs? For example, types of abuse/concerns? (this is from an AE observation that people were presenting/being referred later/further along in the process and of more electronic coercion and using the pandemic as a controlling mechanism)

Have you shared any support or guidance with GPs about how to support patients experience DVA during the pandemic? Did this have an impact?

**Part 2: Engaging with GPs**

Before the pandemic, how did you approach organising and delivering training to GPs?

Has this changed as a result of the pandemic? If so, how?  Has there been any change in the demand or interest in training? Any requests for training for specific issues or questions?

Have you delivered any online training? If yes, how was this?

Has the referral process to IRIS changed during the COVID-19 lockdown and ongoing pandemic restrictions. If yes, how? Has this evolved throughout the pandemic/lockdown phases? Any thoughts on learning throughout this?

In general, how have you found interactions with GPs during the pandemic?

What other support would help GPs to engage with patients about DVA during the pandemic?

**Part 3: Providing support to service users**

Before the pandemic, how did you support patients referred to you who had been affected by DVA?

Has the pandemic changed the way in which you support patients affected by DVA? How? What about professional support and support for yourself and your team?

From your perspective of supporting service users, have you heard any observations about access/pathways to care?

Did you receive any guidance about supporting service users during the pandemic (e.g. from own organisation, from IRISi)? If so, how useful was it?

Have you received support for addressing DVA from any other colleagues or services (e.g. police, social services)

What would enable you to better support service users? – this could include what would help you in your role? Or other support more widely?

**Summary question:**

To summarise from our discussion, what would you say has been the main impact of the pandemic on your work in relation to DVA? On reflection… what changes would you keep? What would you want to change?

**Topic Guide 3 – Practice management and administration team**

**Part 1: General introductory questions**

In general, what role do you think primary care teams play in supporting patients affected by DVA?

Before the pandemic, what role did you have in the practice in relation to DVA?

In general, how has the pandemic impacted day-to-day work in your practice?

How have these changes impacted the support primary care teams are able provide to patients experiencing DVA?

Has your practice adapted or reorganised any processes of work in relation to DVA or safeguarding?

**Part 2: Providing support to patients affected by DVA during the pandemic**

How did you work with the IRIS service to support patients?

Has the pandemic changed the way in which your practice supports patients affected by DVA? How? Do you think this is the same for colleagues across primary care?

Have you received any support or guidance about how to support patients experience DVA during the pandemic? If so, how useful was it?

Has the referral process to IRIS changed during the COVID-19 lockdown and ongoing pandemic restrictions, and how?

In general, how have you found interactions with the IRIS service during the pandemic?

Have you received support for addressing DVA from any other colleagues or services (e.g. police, social services)

What other support would help GPs to engage with patients about DVA?

**Part 3: Training**

Before the pandemic had your practice received training from the IRIS team?

If yes, which training? (e.g. session 1/2/refresher?)

What was the process of organising the training?

How was the training? Did it have an impact on DVA identification/referral?

Have you received any online training from the IRIS team?

If yes, how was this? Were there any practical issues?

Was there anything else you would have liked included?

(if practice has received face-to-face training from IRIS before) How was it different? Was anything better/worse?

**Summary question:**

To summarise from our discussion, what would you say has been the main impact of the pandemic on work in your practice in relation to DVA?
